# Fabrication of Polyaniline/Graphene Oxide Nanosheet@ Tea Waste Granules Adsorbent for Groundwater Purification

**DOI:** 10.3390/nano12213840

**Published:** 2022-10-31

**Authors:** Misfer Al Hawash, Rajeev Kumar, Mohamed A. Barakat

**Affiliations:** Department of Environmental Sciences, Faculty of Meteorology, Environment and Arid Land Agriculture, King Abdulaziz University, Jeddah 21589, Saudi Arabia

**Keywords:** bromide, adsorption, PANI/GO@GTW, kinetics, isotherm, groundwater

## Abstract

The reuse and separation of nanomaterials from an aquatic solution is always challenging and may cause nanotoxicity if not separated completely. Nanomaterial immobilization on the surface of a macro-size material could be an effective approach to developing an efficient composite for groundwater purification. Herein, polyaniline and graphene oxide nanosheet immobilized granular tea waste (PANI/GO@GTW) has been synthesized to remove the anionic and cationic contaminants from groundwater. The synthesized materials were characterized by SEM, XRD, XPS, and FTIR spectroscopies. The optimization of experimental conditions was tested for bromide (Br^−^) removal from synthetic water. The results revealed that Br^−^ adsorption behavior onto the synthesized materials was as follows: PANI/GO < PANI/GTW < PANI < PANI/GO@GTW. The optimum removal of Br^−^ ions was observed at pH 3 with 90 min of saturation time. Br^−^ adsorption onto PANI/GO@GTW followed the pseudo-first-order kinetic and Langmuir isotherm model, and electrostatic interaction was involved in the adsorption process. The optimum adsorption of Br^−^ onto PANI/GO@GTW was found to be 26.80 m/g. The application of PANI/GO@GTW on real groundwater treatment demonstrated the effective removal of anion pollutants such as F^−^, Cl^−^, Br^−^, NO_3_^−^, and PO_4_^3^^−^. This study revealed that PANI/GO@GTW successfully reduced Br^−^ concentrations in synthetic and real groundwater and can be used for large-scale applications.

## 1. Introduction

Groundwater is a vital resource for human beings, animal agriculture, and wildlife. Various water sources, including groundwater, contain different organic and inorganic compounds. Bromide (Br^−^) is the most abundant form of bromine and is present in all fresh water at different concentrations [1]. In addition to natural mechanisms, such as the dissolution of geologic sources and seawater intrusion, anthropogenic activities including chemical production, seawater desalination, plants run by coal, extraction of potassium, and oil and gas production plants may be a factor in increasing bromide concentrations in water sources [2,3,4]. The WHO reports that bromide can be used as an anticonvulsant and sedative at doses of up to 6 g/day. Moreover, elevated doses of bromide cause coma, nausea, paralysis, and vomiting [5]. Normally, oxidative treatment by either chlorination or ozonation is applied to water to disinfect the product and eliminate taste and dour compounds [6]. Nonetheless, both oxidative water approaches result in the formation of non-desired and possibly carcinogenic brominated disinfection by-products (DBPs) such as brominated total trihalomethanes (TTHMs) and bromate $\left({\mathrm{BrO}}_{3}^{-}\right)$ [5,7]. Altogether, removal methodologies aim to control the formation of brominated DBPs [8,9].

Currently, various anion and cation removal strategies are employed, including membrane techniques, photocatalysis, electrochemical methods, adsorption, and coagulation [8,10,11,12,13,14]. Adsorption, in particular, has been regarded as efficient and cost-effective. However, not much research has been conducted on bromide removal. The adsorption of bromide on nanostructured δ-Bi_2_O_3_ was investigated by [15], and results showed that bromide adsorption at a neutral pH and with the absence or low concentrations of other ions would be very effective. The application of silver-immobilized porous carbon for the removal of bromide ions was investigated by Gong et al., 2013 [16]. The prepared silver-immobilized porous carbon showed a poor Br^−^ adsorption capacity (15 µmol/g) for a very long time (28 h). Liu et al. [17] synthesized Fe(III)-complexed carboxylated cellulose beads (Fe-CCBs) for the removal of Br^−^. Adsorption in the latter study occurs through electrostatic attraction and surface complexation. However, the Fe-CCBs showed a low adsorption capacity, 1.224 mg/g for Br^−^ ions. These studies demonstrate that the adsorption of Br^−^ using relatively weak adsorbents is not very effective, and a new strong adsorbent having a high adsorption capacity and low cost must be developed. 

Nowadays, nanomaterials are widely developed and investigated for the application of groundwater purification. A variety of nanomaterials, such as polymers, carbons, metal oxides, etc., show great potential for the pollutant decontamination of wastewater. Graphene oxide (GO) nanosheets are one of the most explored materials in various aspects of science. The presence of multiple functional groups and the sheet-like structure makes them an excellent adsorbent for water purification due to the easy access of the active sites to the pollutants [18]. However, the agglomerative nature of the GO sheets and their separations from the aquatic solution is always challenging [19]. To avoid agglomeration, researchers use GO-based composite adsorbents. However, the separation of the nano adsorbents remains a challenge. The immobilization of the GO sheets on the macro-size materials could be a possible solution that works as a support and enhances Br^−^ adsorption from the solution. Furthermore, the use of agricultural waste could be more suitable due to its low cost and biodegradable properties [20].

The use of tea waste (TW) is recommended as immobilizing support for the GO sheets due to its abundance, ease of handling, and low cost. Moreover, tea waste itself can work as an adsorbent for the removal of Br^−^ due to the presence of functional groups related to its cellulosic or phenolic and flavonoid constituents [21]. The application of tea waste-based adsorbents is already widely explored for the removal of multiple pollutants. Hassan et al. [22] have written a brief review of the application of the TW-based adsorbent. In recent years, TW-based composite adsorbents such as SiO_2_@TW [23], rGO/Fe_3_O_4_/TW [21], Polypyrrole/Magnetic/Tea Waste [24] etc. have been developed for the elimination of diverse pollutants. Based on the water purification application of the TW adsorbents, they can be used as a support to GO sheets and adsorbents to remove Br^−^ from the solution. However, the interaction between the GO sheets and TW might be very strong, GO may be leached into the solution, and nanotoxicity may occur. Therefore, a binder is needed to bind the GO and TW and support the Br^−^ scavenging. A polymeric material such as polyaniline (PANI) could be a suitable binder and promising adsorbent for the anionic Br^−^ ions from the solution due to the positive change in the polymeric moiety [25]. The easy synthesis and low cost are significant advantages of the PANI for its use for environmental remediation applications. 

In this study, polyaniline (PANI) and graphene oxide nanosheets immobilized on a granular tea waste (PANI/GO@GTW) composite have been synthesized using one facile oxidative polymerization approach and used for the decontamination of bromide ions (Br^−^) from groundwater. The adsorption performance of the PANI/GO@GTW was also tested on various anionic and cationic pollutants in the groundwater. The results revealed that the PANI/GO@GTW composite is an efficient material for the decontamination of groundwater. 

## 2. Materials and Methods

All chemicals used in this research were of analytical grade. Used granular black tea leaves were collected locally. Graphite powder used for the GO nanosheet synthesis was obtained from Sigma Aldrich (Saint-Louis, MO, USA). Ammonium persulfate, acetone, and ethanol were procured from Fisher Scientific, Loughborough, UK. Aniline was procured from Sisco Research Laboratories Pvt. Ltd. KBr (Sigma Aldrich) was used to prepare the bromide solution. 

### 2.1. Groundwater Sampling

The real groundwater sample was collected from a water well at Madrakah valley, Makkah region. This water well has been used for several years as a source of water for one of the local bottled drinking water brands. The groundwater sample was collected in sterilized glass bottles. Samples were then stored at 4 °C and analyzed using ICP within two days of collection. 

### 2.2. Synthesis of Polyaniline/Graphene Oxide@ Granular Tea Waste

Granular Tea waste (GTW) was washed with hot and cold de-ionized water several times to remove soluble substances. After, the washed GTW was dried at 80 °C for 24 h and stored for further use. Graphene oxide nanosheets were synthesized using the slightly modified Hummer’s methods using graphite powder. A detailed process of GO synthesis is reported elsewhere [26]. 

Here, PANI/GO@GTW was synthesized via oxidation polymerization of the aniline in the presence of the GO nanosheets and GTW. Initially, 20 mL of GO solution was dissolved in 60 mL of 0.25 M HCL solution. After that, 1.4 g of GTW was suspended in the GO solution and continuously stirred for 30 min in an ice bath. Then, 3.1 mL of aniline was added and stirred for 30 min. Ammonium persulfate solution was prepared in 0.25 M of HCl. This solution was then added dropwise to polymerize the aniline on the surface of the GO/GTW. The mixture was stirred for 18 h, followed by a filtration step to remove the green precipitate, and finally washed with plenty of de-ionized water to purify the PANI/GO@GTW composite. The PANI/GO@GTW composite was washed with acetone and ethanol until the color was washed out. The obtained PANI/GO@GTW composite was dried at 105 °C for 12 h. A similar method was used to synthesize the pure PANI, PANI@GTW, and GO/PANI nanocomposite. 

### 2.3. Characterization

X-ray diffraction (XRD) analysis was done by an ULTIMA IV X-ray diffractometer (Manufactured by Rigaku, Tokyo, Japan) in a continuous 2Theta/Theta scanning mode at a current of 40 mA in the scanning range of 5–90°. The morphology of the GTW and PANI/GO@GTW were recorded on a Scanning Electron Microscope (SEM) (JEOL-JSM7600F, Tokyo, Japan). The FTIR spectrum of GTW and PANI/GO@GTW before and after Br^−^ were analyzed on a Thermo Is50 spectrophotometer. In addition, X-ray Photoelectron spectroscopic analysis (XPS was performed on a Specs GmbH (Specs GmbH, Berlin, Germany) X-ray Photoelectron spectrophotometer. The chemical analysis of anions in the groundwater samples before and after adsorption was done using an Inductively Coupled Plasma-Optical Emission Spectrometer (ICP) and Ion Chromatographer (IC) analysis. The ICP used was supplied by Agilent Technologies, Palo Alto, CA, USA, model: 720 ICP-OES Axial. The IC used was provided by Metrohm, model: 850 Professional IC, Herisau, Switzerland.

### 2.4. Br^−^ Adsorption Experiments

Adsorption of the bromide onto the synthesized materials was initiated by adding 0.05 g of the materials into 20 mL of the bromide-containing solution under continuous shaking at 200 rpm. The adsorption of Br^−^ ions onto the pure PANI, PANI@GTW, PANI/GO, and PANI/GO@GTW at different solution pHs (3 to 9) was studied, the solution’s pH was modified using 0.1 M of HCl and NaOH, at 30 °C for a 3 h interaction time. The Br^−^ concentration effect on the adsorption was investigated in the range from 1 to 120 mg/L at pH 3. The active site saturation time of PANI/GO@GTW was investigated in the range of 0 to 150 min. The amount of the Br^−^ scavenged for the solution was analyzed by an Ion Chromatographer (IC). The adsorption capacity was determined using the following formula:

q = (C_i_ − C_e_) × V/m (1)

where q is Br^−^ adsorption capacity (mg/g), C_i_ is the initial Br^−^ concentration (mg/L), C_e_ is the Br^−^ concentration at adsorption equilibrium (mg/L), V is the volume of Br^−^ solution (L), and m is the weight of the adsorbents (g). 

## 3. Results and Discussion

### 3.1. Synthesis and Characterization

**Figure 1:**
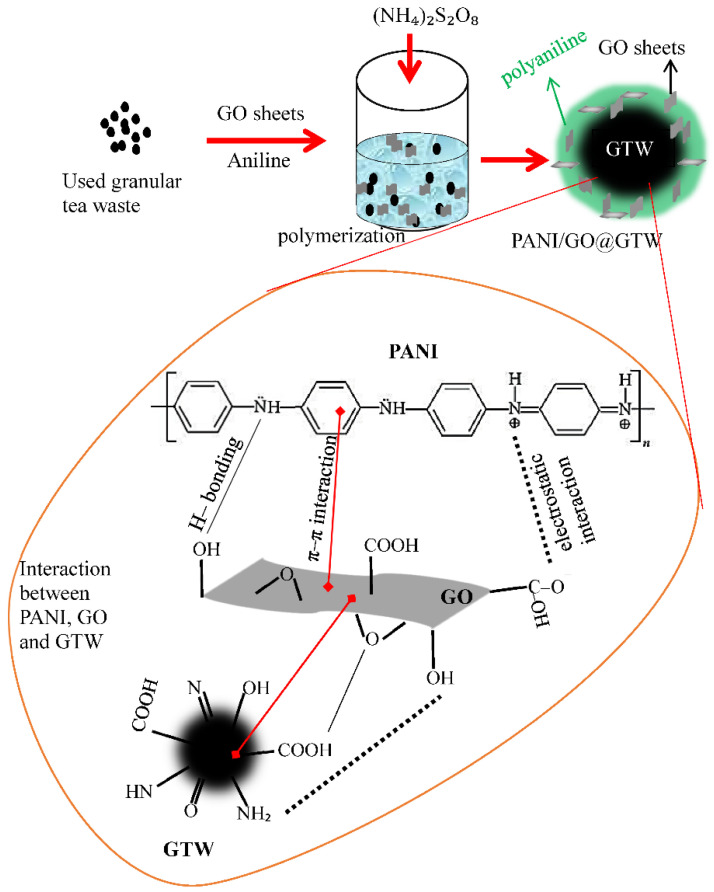
Schematic diagram for the synthesis of the PANI/GO@GTW composite.

A facile oxidation method was used to synthesize the PANI/GO@GTW composite. The used GTW is the core material. having functional groups such as COOH, NH_2_, NH, OH, C = C, etc. from the various components of the tea leaves such as cellulose, lignin, amino acids, pectin, carbohydrate, polyphenols, etc. [22], while the GO sheets have the C = C, COOH, OH, = O, etc. groups and polyaniline has the NH, NH_2_, and C = C groups on their surface. These functional groups present on the surface of the GTW, GO, and PANI can interact through H-bonding, π–π, and electrostatic bonding [27]. A schematic diagram for synthesizing the PANI/GO@GTW composite has been shown in Figure 1. 

**Figure 2:**
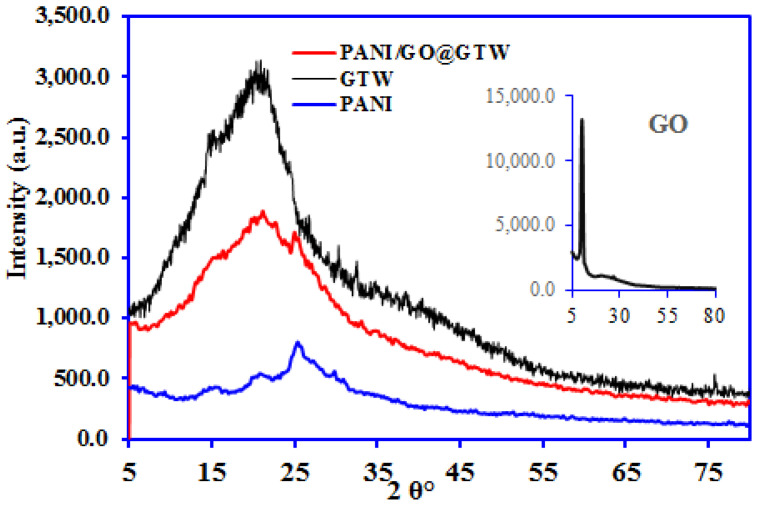
XRD pattern of the GTW, GO (inset), PANI, and PANI/GO@GTW composite.

Various characterization tools have been used to confirm the successful synthesis of the prepared composite materials. Figure 2 depicts the XRD pattern of the pure GO, PANI, GTW, and PANI/GO@GTW composites. The XRD pattern of the GO reveals a characteristic peak at 10.23° (001), confirming the successful conversion of the graphite to GO. The XRD pattern of the GTW demonstrates a wide peak centered at 21° due to the cellulosic structure, indicating the amorphous nature [28]. The PANI shows the semicrystalline nature as the peaks appeared at 21.34° and 26.69° [29]. The PANI/GO@GTW composite pattern is similar to GTW and PANI, having the same peaks for the cellulosic structure in tea and PANI at 21.25° and 26.2°. However, the characteristic peak for the GO was not observed in PANI/GO@GTW due to its low amount. Moreover, the amorphous nature of the GTW may hide the peak for the GO in the PANI/GO@GTW composite. 

**Figure 3:**
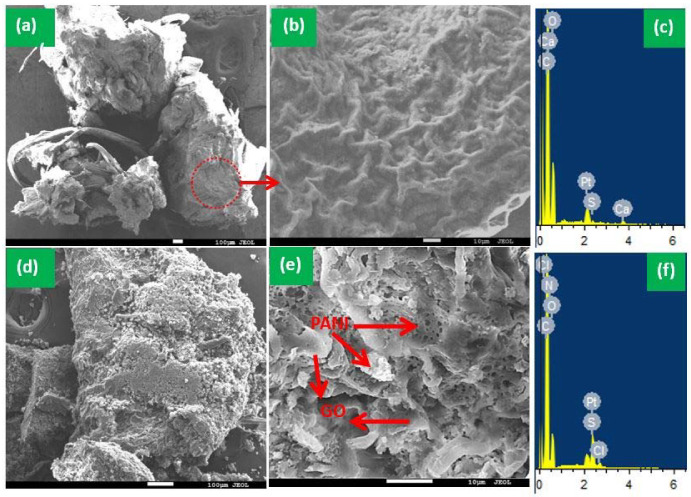
SEM images and EDX analysis of (**a**–**c**) granular tea waste and (**d**–**f**) PANI/GO/@GTW.

The surface morphology and EDX analysis were performed to examine the surface texture and any changes in the elemental composition after the immobilization of the PANI and GO sheets on the surface of the GTW composite. The SEM images (Figure 3a,b) show the irregular granules of the rough surface of GTW, and the C, O, Ca, and S elements were observed by EDX analysis (Figure 3c). After the PANI and GO immobilization on the GTW surface, the adherence of small particles can be seen (Figure 3d). The magnified image of the PANI/GO@GTW composite showed the porous structure of the PANI, GTW, and GO sheets are visible. The elements on the PANI/GO@GTW composite surface were observed as C, O, N, Cl, and S. The Pt presences in both materials were due to the Pt coating before the SEM analysis. Moreover, Ca was not observed in the PANI/GO@GTW composite, probably due to its solubilization in the presence of HCl at the synthesis time. 

**Figure 4:**
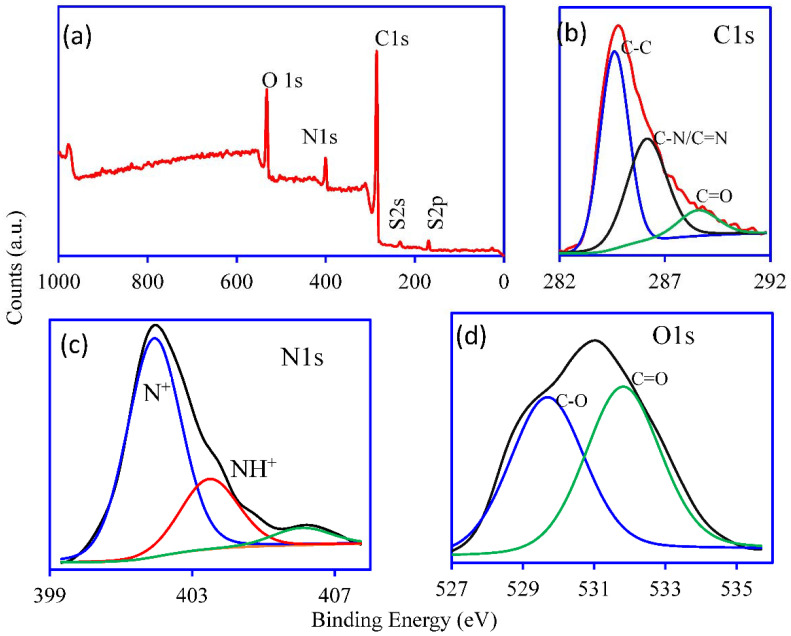
XPS analysis of the PANI/GO@GTW (**a**) wide scan survey and deconvoluted spectra of (**b**) C1s, (**c**) N1s, and (**d**) O1s.

The identification of elemental compositions and their oxidation states was done using the XPS technique. The wide scan survey of the PANI/GO@GTW composite is shown in Figure 4a, indicating the presence of the C—75.7%, O—15.4%, N—7.6%, and S—1.2% for the C1s, O1s, N1s, and S2s, respectively. The deconvolution of the C1s (Figure 4b) indicates the presence of the C-C (184.33 eV), C-N/C = N (286.12 eV), and C = O (289.14 eV) groups which belong to the GO, PANI, and GTW [30]. Figure 4c indicates that several peaks for the deconvoluted N1s correspond to N^+^ (401.7 eV), and NH^+^ (403.1 eV) [31]. The XPS spectra of O1s showed the two peaks at 529.68 eV and 531.8 eV, indicating the presence of C-O and C = O related groups in the GO and GTW as shown in Figure 4d [32]. The XPS analysis confirmed the surface elements of the PANI/GO@GTW composite that are present in the synthesized material. 

**Figure 5:**
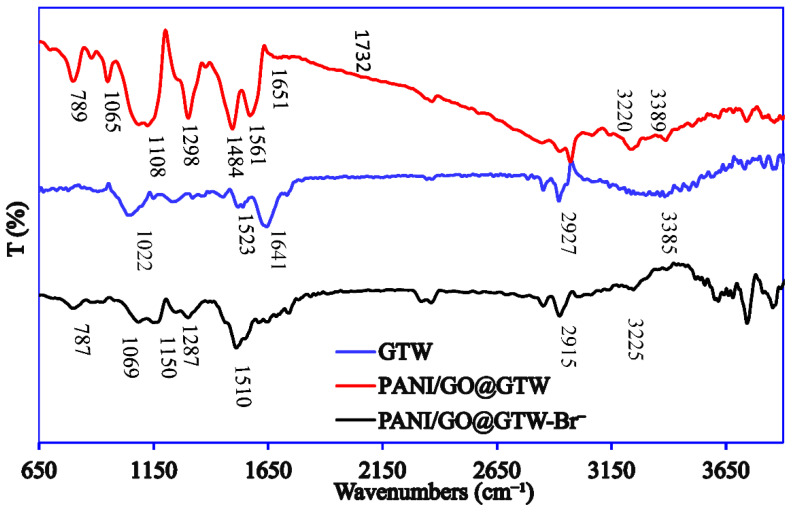
FTIR spectrum of GTW, PANI/GO@GTW, and Br^−^ adsorbed PANI/GO@GTW.

The FTIR analysis of the GTW, PANI/GO@GTW, and Br^−^ adsorbed PANI/GO@GTW is included in Figure 5. The FTIR spectrum of the pure GTW shows several absorption peaks for aliphatic C-H groups (2917 and 2844 cm^−1^), C = O (1641 cm^−1^), and CH_3_ symmetric bending (1523 cm^−1^). The absorption peaks at 1442, 1202, 1125, and 1022 cm^−1^ suggest the presence of the NH_2_, CH_2_, C-O, and C = O groups in the pectin, cellulose, and lignin, etc. moieties in the tea waste [33]. After the PANI and GO nanosheet immobilization, the FTIR spectrum of the PANI/GO@GTW and GTW showed a similar pattern along some new characteristic peaks of PANI and GO. The broad peaks that appear at 1561, 1484, and 1108 cm^−1^ are the PANI benzene rings, while the peaks at 1294 and 789 cm^−1^ correspond to C-N stretching and the out-of-plan vibration of C-H [31]. The peaks for the GO in the PANI/GO@GTW were observed at 1651 (C = C stretching) and 1065 (C-O stretching) [34]. The FTIR spectrum of the Br^−^ adsorbed PANI/GO@GTW showed a similar pattern to the PANI/GO@GTW spectra. A change in the peak position and peak intensity of the –OH, -C = O, -N-H, and -C-C- is observed after the Br^−^ adsorption onto PANI/GO@GTW. These results suggest that the change in the peak position and peak intensity is owed to the interaction between Br^−^ ions and diverse functional groups found on the surface of PANI/GO@GTW [33].

### 3.2. Removal of Br^−^ Adsorption

The adsorption of the Br^−^ onto the synthesized material has been investigated under the influence of a number of operations confines such as the pH of the solution, interaction time between the Br^−^ and synthesized materials, and Br^−^ concentration in the solution.

**Figure 6:**
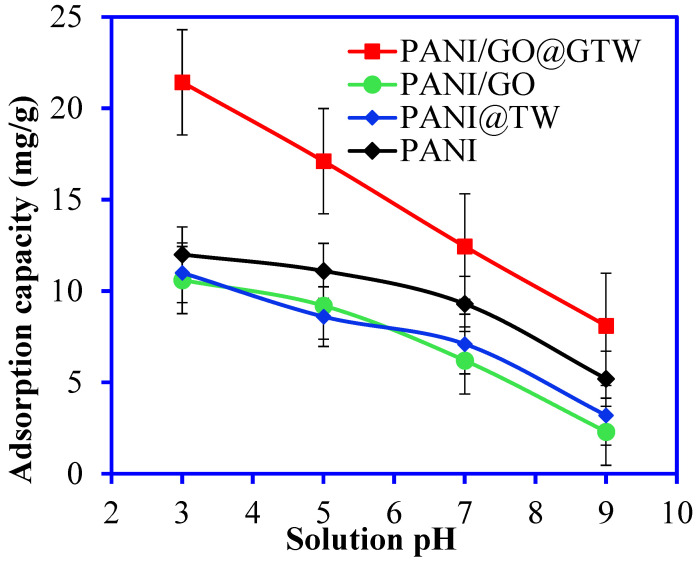
Adsorption of the Br^−^ ions from aqueous solution at various solution pHs (Br^−^ conc.: 50 mg/L, time: 150 min, solution volume: 20 mL, adsorbent mass: 0.05 g).

**Figure 7:**
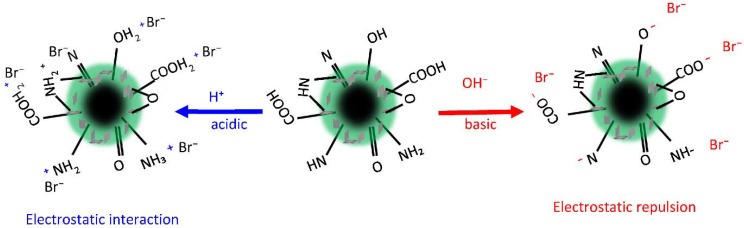
Schematic representation of the Br^−^ adsorption mechanism of PANI/GO@GTW under acidic and basic solution conditions.

The solution pH is instrumental in Br^−^ adsorption as it strongly overlaps with hydrogen desorption, particularly in an acidic medium [35]. Figure 6 demonstrates the interaction behavior of the pure PANI, PANI/GTW, PANI/GO, and PANI/GO@GTW. The Br^−^ adsorption results indicate that the PANI/GO@GTW composite is the most efficient adsorbent, and the following Br^−^ adsorption behavior is observed: PANI/GO < PANI/GTW < PANI, <PANI/GO@GTW. The PANI/GO showed a lower adsorption capacity, possibly due to the convergence of the GO active sites by the PANI. In this situation, the GO active sites were not available for Br^−^ adsorption. The highest Br^−^ adsorption was observed in the acidic medium. The adsorption behavior of the Br^−^ ions onto the prepared materials can be owed to the surface functional groups on the PANI/GO@GTW composite. The presence of these various functional groups on the surface facilitates l more Br^−^ ion adsorption. Therefore, the PANI/GO@GTW composite showed the highest Br^−^ adsorption at all the studied pHs than the PANI, PANI/GTW, and PANI/GO composites. Although pH 3 is the most suitable condition for the adsorption of the anionic Br^−^ ions, due to electrostatic interaction with the positively charged adsorbent surface, the adsorbent surface absorbed the H^+^ from the solution, and simultaneously, Br^−^ ions co-adsorbed to compensate for the surface charge [36]. With the rise in the solution temperature, the −OH ion concentration increased, which is a competitor of the Br^−^ ions for the same active sites [17]. Moreover, excess −OH ions cause the deprotonation of the amine, carboxylic, and hydroxylic groups, resulting in electrostatic repulsion between the negatively charged adsorbent surface and Br^−^ ions [37]. Hence, a gradual reduction in Br^−^ adsorption was observed at higher pH conditions. A schematic diagram for the adsorption of Br^−^ ions onto the PANI/GO@GTW composite is shown in Figure 7 under acidic and basic solution conditions. Based on this study, pH 3 and the PANI/GO@GTW composite were selected for further optimization of the experimental conditions. 

**Figure 8:**
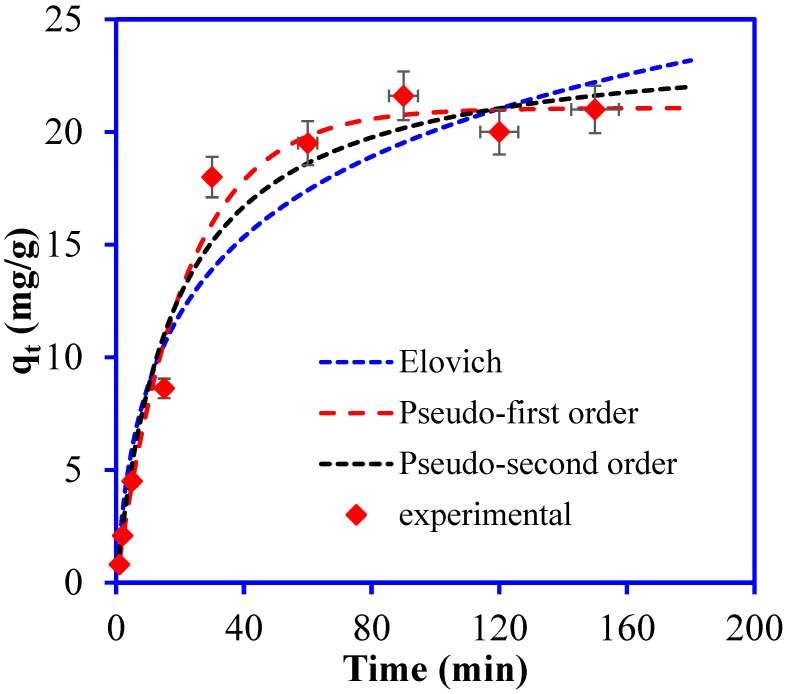
Kinetics plots for the adsorption of Br^−^ ions onto PANI/GO@GTW from aqueous solution (solution pH: 3, Br^−^ conc.: 50 mg/L, time: 150 min, solution volume: 20 mL, and adsorbent mass: 0.05 g).

The Br^−^ adsorption studies were performed at different time intervals to optimize the PANI/GO@GTW active site saturation time (from 0–150 min). Equilibrium of the adsorption rate was reached quickly and signified a slight variation from 60 min to 120 min. However, the highest Br^−^ adsorption capacity (21.1 mg/g) was recorded at 90 min, as illustrated in Figure 8. The Br^−^ binding at the vacant sites of PANI/GO@GTW was fast, and most Br^−^ ions adsorbed within 30 min, with adsorption increasing gradually with the complete saturation of the active sites [38].

The adsorption kinetics of Br^−^ on PANI/GO@GTW was studied using pseudo-first-order and pseudo-second-order, Elovich kinetic models. The linearized equations for the applied models are as follows:

(2) $$\mathrm{Pseudo-first}\;\mathrm{order}:\;q_{t}=\mathrm{qe}\left(1-e^{\left(-k_{1}t\right)}\right)$$

(3) $$\mathrm{Pseudo-second}\;\mathrm{order}:\;q_{t}=\frac{q_{e}^{2}k_{2}t}{\left[k_{2}\left(q_{e}\right)t+1\right]}$$

(4) $$\mathrm{Elovich}\;\mathrm{model}:\;\;q_{t}=\frac{1}{\beta }\ln\left({\alpha \beta }_{t}\right)$$

where, q_e_ and q_t_: Br^−^ adsorption capacities at equilibrium and at time t (min), k_1_: Pseudo-first order rate constant, k_2_: Pseudo-second order rate constant, and α and β: adsorption and desorption rate constants for the Elovich model. The Br^−^ adsorption kinetics plots are shown in Figure 8. The values of the kinetic parameters calculated for the plots are tabulated in Table 1. The experimental Br^−^ adsorption capacity and calculated Br^−^ adsorption capacity for the pseudo-first-order kinetic model are close compared to the estimated Br^−^ adsorption capacity for the pseudo-second-order kinetic. Moreover, the R^2^ value for the pseudo-first-order kinetic (R^2^: 0.984) is higher than the pseudo-second-order (R^2^: 0.970) and Elovich model (R^2^: 0.938). The value of statistical parameter χ^2^ (chi-square) is low and the RMSD (root-mean-square deviation) value is high for the pseudo-first-order kinetic model. These results reveal that the Br^−^ adsorption on the PANI/GO@GTW composite follows the pseudo-first-order kinetic model, and the rate change in Br^−^ ions’ uptake with time is directly proportional to the difference in saturation concentration and solid uptake with time [39].

**Figure 9:**
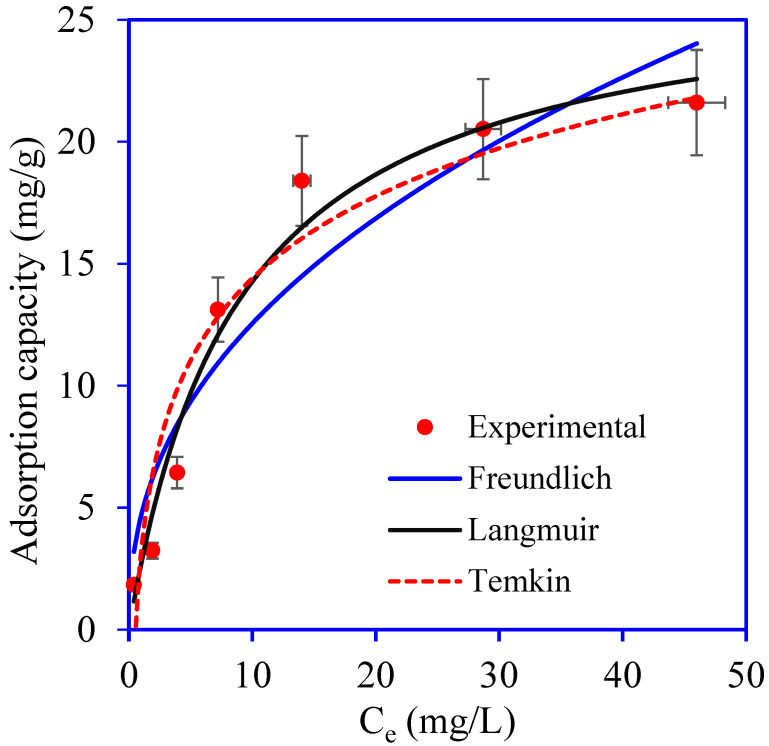
Adsorption isotherm plots for the adsorption of Br^−^ ions onto PANI/GO@GTW from aqueous solution. (solution pH: 3, solution volume: 20 mL, and adsorbent mass: 0.05 g).

The Br^−^ ion concentration in the solution is a key player in adsorption. Herein, the effect of varying Br^−^ ion concentrations was explored in the range from 1–120 mg/L at pH 3. The adsorption capacity of PANI/GO@GTW for Br^−^ increased from 1.84 to 21.7 mg/g with the change in the concentration from 1 to 120 mg/g (Figure 9). The adsorption of Br^−^ at lower concentrations was low due to the lower number of Br^−^ ions and a higher number of active sites on PANI/GO@GTW. As the concentration of Br^−^ ions increases in the solution, the difference between the Br^−^ ions and available active sites reduces. Therefore, all active sites on PANI/GO@GTW occupied by Br^−^ ions at higher concentrations resulted in equilibrium attainment. Moreover, increasing the Br^−^ ions increases the driving force, which is sufficient to overcome the mass transfer resistance between the solid and liquid phases [38].

Data from experimental Br^−^ adsorption trials were examined using the Freundlich, Langmuir, and Temkin isotherm equations to predict the interaction between Br^−^ ions and PANI/GO@GTW. The applied models’ equations are depicted in Table 2. Figure 9 shows the plots for the used isotherm models, and the values of the isotherm parameters are summarized in Table 2. The maximum monolayer adsorption capacity of the PANI/GO@GTW for Br^−^ was observed at about 26.92 mg/g. The values of R^2^ (0.972), RMSE (1.29), and χ^2^ (1.62) better fit the Langmuir isotherm model compared to the Freundlich (R^2^: 0.901, RMSE: 2.427, and χ^2^: 4.230) and Temkin (R^2^: 0.910, RMSE: 2.306, and χ^2^: 4.587) isotherm models. The isotherm analysis results revealed that the Br^−^ adsorption onto PANI/GO@GTW follows the Langmuir isotherm and monolayer adsorption occurs as each site binds with a single Br^−^ ion [16].

### 3.3. Regeneration and Reusability Studies 

**Figure 10:**
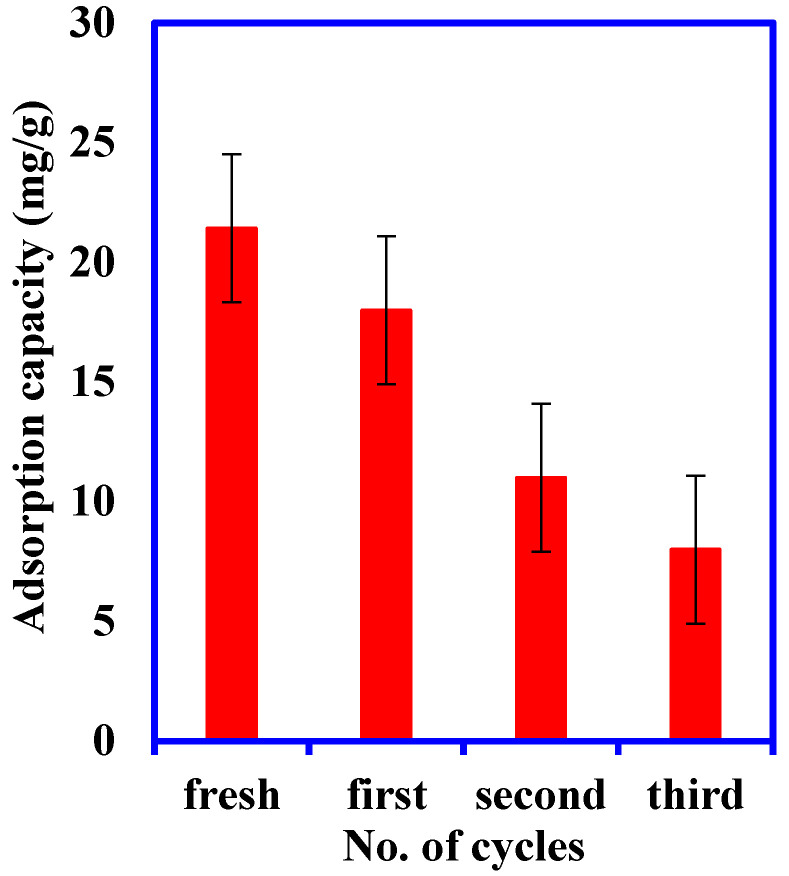
Analysis of the reusability and adsorption efficiency of the spent PANI/GO@GTW.

This is an additional step conducted to evaluate the ability of the PANI/GO@GTW to adsorb the Br^−^ ions over a number of cycles. The reuse of the PANI/GO@GTW started with washing the leftover saturated materials with acetone and ethanol. As shown in Figure 10, there was a decrease in the adsorption capacity from 21.1 mg/g to 8 mg/g after four cycles. It can be assumed that all the active sites were not regenerated during the desorption process; therefore, the reusability of the PANI/GO@GTW decreases gradually [17]. 

### 3.4. Real Groundwater Treatment

**Figure 11:**
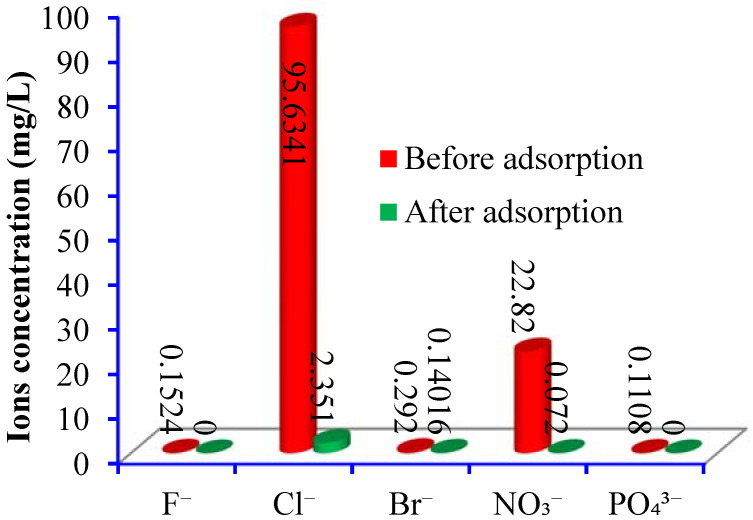
Groundwater decontamination properties of the PANI/GO@GTW.

The efficacy of the PANI/GO@GTW was tested for the removal of Br^−^ ions from real groundwater. Groundwater commonly consists of several dissolved anions and cations; the IC analysis of the dissolved anions is shown in Figure 11, indicating the presence of F^−^, Cl^−^, Br^−^, NO_3_^−^, and PO_4_^3−^ in the groundwater. Generally, co-existing ions compete for the same active site in the adsorption process. The adsorption efficacy of the PANI/GO@GTW composite for the various anions is shown in Figure 11. The results demonstrated that F^−^ and PO_4_^3−^ are completely removed from the groundwater and the concentration of the Cl^−^, Br^−^, and NO_2_^−^ is within the permissible limit. These results revealed that PANI/GO@GTW is an efficient material for scavenging the anions from real groundwater. 

### 3.5. Comparison of the Adsorption Capacities

The adsorption efficiencies of diverse adsorbents used to remove Br^−^ ions are demonstrated in Table 3. The adsorption of the Br^−^ ions depends to a large extent on the experimental conditions. The values in Table 3 demonstrate that PANI/GO@GTW is much more efficient than most of the previously reported adsorbents. 

## 4. Conclusions

In this study, polyaniline and graphene oxide nanosheet immobilized granular tea waste (PANI/GO@GTW) was successfully synthesized as a new adsorbent for the removal of Br^−^ from groundwater. The optimization of the adsorption was investigated under different parameters, including the effects of pH, initial concentration, and contact time. The findings of this study revealed that Br^−^ removal was best at pH 3, with a tendency to favor an acidic medium. The adsorption kinetic data were fitted to the pseudo-first-order, pseudo-second-order, and Elovich models, while the Langmuir, Freundlich, and Temkin isotherm models were fitted to the Br^−^ equilibrium data. Based on the statistical analysis, root-mean-square deviation (RMSD), χ^2^, and R^2^ values, the pseudo-first-order model and Langmuir isotherms were the best fitted models. The maximum monolayer adsorption was found to be 26.8 mg/g. The isotherm analysis results revealed that all the active sites are energetically equivalent and Br^−^ formed a monolayer on the surface of the PANI/GO@GTW. The groundwater treatment results revealed that F^−^ and PO_4_^3−^ are completely removed, while the concentration of the Cl^−^, Br^−^, and NO_2_^−^ were reduced to a permissible limit. These results indicate that PANI/GO@GTW is highly efficient in cleaning pollutants from groundwater. 

## Figures and Tables

**Table 1:** Adsorption kinetics parameters for Br^−^ onto GO/PANI@GTW.

Kinetic Model	Parameters	Values
Pseudo-first-order:	qe (exp) (mg g^−1^):	21.1
	qe (cal) (mg g^−1^):	21.062
	k_1_ (min^−1^):	0.047
	R^2^:	0.984
	RMSE:	1.016
	χ^2^	0.796
Pseudo-second-order:	qe (cal) (mg g^−1^):	24.210
	k_2_ (g mg^−1^ min^−1^):	0.0022
	R^2^:	0.970
	RMSE:	1.419
	χ^2^	1.650
Elovich model:	a (mg g^−1^ min^−1^):	2.196
	β (mg g^−1^):	0.186
	R^2^:	0.938
	RMSE:	2.027
	χ^2^	3.717

where, χ^2^: chi-square and RMSD: root-mean-square deviation.

**Table 2:** Adsorption isotherm parameters for Br^−^ onto GO/PANI@GTW.

Isotherm Model	Parameters	Values
Langmuir $q_{e}=\frac{q_{m}k_{L}C_{e}}{1+k_{L}C_{e}}$	q_m_ (mg g^−1^):	26.920
	K_L_ (L mg^−1^):	0.112
	R^2^:	0.972
	RMSE:	1.290
	χ^2^:	1.620
Freundlich $q_{e}=k_{F}C_{e}{}^{\frac{1}{n}}$	n:	2.351
	K_f_ (mg g^−1^) (mg L^−1^)^−1^/nF:	4.717
	R^2^:	0.901
	RMSE:	2.427
	χ^2^:	4.230
Temkin $q_{e}=B_{t}\;\ln\left(K_{t}C_{e}\right)$	B_t_ (J mg^−1^)	509.409
	K_t_ (L mg^−1^):	1.949
	R^2^:	0.9108
	RMSE:	2.306
	χ^2^:	4.587

where, C_e_: equilibrium concentration, q_e_: equilibrium capacity, q_m_: monolayer adsorption capacity, k_F_ and 1/n: Freundlich constant related to adsorption intensity and adsorption capacity, K_t_: equilibrium binding constant, and B_t_: the heat of adsorption.

**Table 3:** Comparison of the adsorption capacities of various materials for Br^−^ removal.

Materials	Efficiency	Conditions						Ref.
		pH	Mass (g)	Time (min.)	Temp. (°C)	Conc. (mg/L)	Volume (mL)	
MIEX resin	11.51 mg/g	4–9	1	60	30	1000	1000	[10]
δ-Bi_2_O_3_	147.1 mg/g	7	2	1440	25	50–500	50	[12]
Purolite-Br	90%	6.5	-	15	21	250	600	[37]
Silver-impregnated activated carbon	93%	6–7	25	170	20	300	1000	[38]
AgCl-activated carbon	>90%	6.5	0.01	2.5	25	1	-	[39]
Granular activated carbon	91.2%	-	400	2–5	-	50	-	[40]
Carbonized xerogel	21%	5	0.02	-	25	10–100	50	[41]
GO/PANI@GTW	29.2 mg/g	3	0.05	90	30	100	20	This study

## Data Availability

All the data include in the Article or available on demand.

## References

[1] Schoonen M.A., Devoe V., Brown C.J. (1995). Bromide in Long Island Groundwaters and Surface Waters.

[2] Magazinovic R.S., Nicholson B.C., Mulcahy D.E., Davey D.E. (2004). Bromide levels in natural waters: Its relationship to levels of both chloride and total dissolved solids and the implications for water treatment. Chemosphere.

[3] Weinberg H.S., Krasner S.W., Richardson S.D., Thruston A. (2002). The Occurrence of Disinfection by-Products (DBPs) of Health Concern in Drinking Water: Results of a Nationwide DBP Occurrence Study.

[4] Von Gunten U. (2003). Ozonation of drinking water: Part II. Disinfection and by-product formation in presence of bromide, iodide or chlorine. Water Res..

[5] World Health Organization (2009). Bromide in Drinking-Water: Background Document for Development of WHO Guidelines for Drinking-Water Quality.

[6] Sedlak D.L., von Gunten U. (2011). The chlorine dilemma. Science.

[7] Aljundi I.H. (2011). Bromate formation during ozonation of drinking water: A response surface methodology study. Desalination.

[8] Watson K., Farré M.J., Knight N. (2012). Strategies for the removal of halides from drinking water sources, and their applicability in disinfection by-product minimisation: A critical review. J. Environ. Manag..

[9] Majidnia Z., Idris A. (2015). Photocatalytic reduction of iodine in radioactive waste water using maghemite and titania nanoparticles in PVA-alginate beads. J. Taiwan Inst. Chem. Eng..

[10] Ateia M., Erdem C.U., Ersan M.S., Ceccato M., Karanfil T. (2019). Selective removal of bromide and iodide from natural waters using a novel AgCl-SPAC composite at environmentally relevant conditions. Water Res..

[11] Rajaeian B. (2017). Removal of Bromide from Drinking Water Sources using Silver Impregnated Activated Carbon (SIAC): Understanding Br-SIAC Interactions. Ph.D. Thesis.

[12] Zhao W., Dong Q., Sun C., Xia D., Huang H., Yang G., Wang G., Leung D.Y. (2021). A novel Au/g-C3N4 nanosheets/CeO_2_ hollow nanospheres plasmonic heterojunction photocatalysts for the photocatalytic reduction of hexavalent chromium and oxidation of oxytetracycline hydrochloride. Chem. Eng. J..

[13] Mu F., Miao X., Cao J., Zhao W., Yang G., Zeng H., Li S., Sun C. (2022). Integration of plasmonic effect and S-scheme heterojunction into gold decorated carbon nitride/cuprous oxide catalyst for photocatalysis. J. Clean. Prod..

[14] Zhao W., Ma S., Yang G., Wang G., Zhang L., Xia D., Huang H., Cheng Z., Xu J., Sun C. (2021). Z-scheme Au decorated carbon nitride/cobalt tetroxide plasmonic heterojunction photocatalyst for catalytic reduction of hexavalent chromium and oxidation of Bisphenol A. J. Hazard. Mater..

[15] Shi M., Guo C., Li J., Li J., Zhang L., Wang X., Ju Y., Zheng J., Li X. (2017). Removal of bromide from water by adsorption on nanostructured δ-Bi2O3. J. Nanosci. Nanotechnol..

[16] Gong C., Zhang Z., Qian Q., Liu D., Cheng Y., Yuan G. (2013). Removal of bromide from water by adsorption on silver-loaded porous carbon spheres to prevent bromate formation. Chem. Eng. J..

[17] Liu S., Cheng G., Xiong Y., Ding Y., Luo X. (2020). Adsorption of low concentrations of bromide ions from water by cellulose-based beads modified with TEMPO-mediated oxidation and Fe (III) complexation. J. Hazard. Mater..

[18] Thakur K., Kandasubramanian B. (2019). Graphene and graphene oxide-based composites for removal of organic pollutants: A review. J. Chem. Eng. Data.

[19] Ghulam A.N., Dos Santos O.A., Hazeem L., Backx B.P., Bououdina M., Bellucci S. (2022). Graphene Oxide (GO) Materials—Applications and Toxicity on Living Organisms and Environment. J. Funct. Biomater..

[20] Li B., Gan L., Owens G., Chen Z. (2018). New nano-biomaterials for the removal of malachite green from aqueous solution via a response surface methodology. Water Res..

[21] Yang A., Zhu Y., Li P., Huang C. (2019). Preparation of a magnetic reduced-graphene oxide/tea waste composite for high-efficiency sorption of uranium. Sci. Rep..

[22] Hussain S., Anjali K., Hassan S.T., Dwivedi P.B. (2018). Waste tea as a novel adsorbent: A review. Appl. Water Sci..

[23] Joshi S., Kataria N., Garg V., Kadirvelu K. (2020). Pb^2+^ and Cd^2+^ recovery from water using residual tea waste and SiO_2_@ TW nanocomposites. Chemosphere.

[24] Saleem F., Bhatti H.N., Khan A., Farhat L.B., Elqahtani Z.M., Alwadai N., Iqbal M. (2022). Polypyrrole/Magnetic/Tea Waste Composites for PO43− Ions Removal: Adsorption-Desorption, Kinetics, and Thermodynamics Studies. Adsorpt. Sci. Technol..

[25] Okhay O., Tkach A. (2022). Synergetic Effect of Polyaniline and Graphene in Their Composite Supercapacitor Electrodes: Impact of Components and Parameters of Chemical Oxidative Polymerization. Nanomaterials.

[26] Jilani A., Hussain S.Z., Ansari M.O., Kumar R., Dustgeer M.R., Othman M.H.D., Barakat M., Melaibari A.A. (2021). Facile synthesis of silver decorated reduced graphene oxide@ zinc oxide as ternary nanocomposite: An efficient photocatalyst for the enhanced degradation of organic dye under UV–visible light. J. Mater. Sci..

[27] Majumdar D. (2016). Functionalized-graphene/polyaniline nanocomposites as proficient energy storage material: An overview. Innov. Energy Res..

[28] Cai H.-M., Chen G.-J., Peng C.-Y., Zhang Z.-Z., Dong Y.-Y., Shang G.-Z., Zhu X.-H., Gao H.-J., Wan X.-C. (2015). Removal of fluoride from drinking water using tea waste loaded with Al/Fe oxides: A novel, safe and efficient biosorbent. Appl. Surf. Sci..

[29] Zhang Y., Liu J., Zhang Y., Liu J., Duan Y. (2017). Facile synthesis of hierarchical nanocomposites of aligned polyaniline nanorods on reduced graphene oxide nanosheets for microwave absorbing materials. RSC Adv..

[30] Tong Z., Yang Y., Wang J., Zhao J., Su B.-L., Li Y. (2014). Layered polyaniline/graphene film from sandwich-structured polyaniline/graphene/polyaniline nanosheets for high-performance pseudosupercapacitors. J. Mater. Chem. A.

[31] Li Y., Xia Z., Gong Q., Liu X., Yang Y., Chen C., Qian C. (2020). Green synthesis of free standing cellulose/graphene oxide/polyaniline aerogel electrode for high-performance flexible all-solid-state supercapacitors. Nanomaterials.

[32] Nagajyothi P.C., Yoo K., Ramaraghavulu R., Shim J. (2021). Hydrothermal Synthesis of MnWO4@ GO Composite as Non-Precious Electrocatalyst for Urea Oxidation. Nanomaterials.

[33] Ebrahimian A., Saberikhah E. (2013). Biosorption of methylene blue onto Foumanat tea waste: Equilibrium and thermodynamic studies. Cellul. Chem. Technol..

[34] Rong X., Qiu F., Zhang C., Fu L., Wang Y., Yang D. (2015). Preparation, characterization and photocatalytic application of TiO_2_–graphene photocatalyst under visible light irradiation. Ceram. Int..

[35] Mello G.A., Briega-Martos V., Climent V., Feliu J.M. (2018). Bromide Adsorption on Pt (111) over a Wide Range of pH: Cyclic Voltammetry and CO Displacement Experiments. J. Phys. Chem. C.

[36] Nishi M., Ohkubo T., Yamasaki M., Takagi H., Kuroda Y. (2017). Surplus adsorption of bromide ion into π-conjugated carbon nanospaces assisted by proton coadsorption. J. Colloid Interface Sci..

[37] Sikdar D., Goswami S., Das P. (2020). Activated carbonaceous materials from tea waste and its removal capacity of indigo carmine present in solution: Synthesis, batch and optimization study. Sustain. Environ. Res..

[38] Kumbhar P., Narale D., Bhosale R., Jambhale C., Kim J.-H., Kolekar S. (2022). Synthesis of tea waste/Fe3O4 magnetic composite (TWMC) for efficient adsorption of crystal violet dye: Isotherm, kinetic and thermodynamic studies. J. Environ. Chem. Eng..

[39] Ifelebuegu A., Ukpebor J., Obidiegwu C., Kwofi B. (2015). Comparative potential of black tea leaves waste to granular activated carbon in adsorption of endocrine disrupting compounds from aqueous solution. Glob. J. Environ. Sci. Manag..

[40] Zhang Y.-Q., Wu Q.-P., Zhang J.-M., Yang X.-H. (2015). Removal of bromide and bromate from drinking water using granular activated carbon. J. Water Health.

[41] Medellin-Castillo N.A., Isaacs-Páez E.D., Giraldo-Gutierrez L., Moreno-Piraján J.C., Rodríguez-Méndez I., Reyes-López S.Y., Reyes-Hernández J., Segovia-Sandoval S.J. (2022). Data for the synthesis, characterization, and use of xerogels as adsorbents for the removal of fluoride and bromide in aqueous phase. Data Brief.

